# Genetic basis of defects in immune tolerance underlying the development of autoimmunity

**DOI:** 10.3389/fimmu.2022.972121

**Published:** 2022-08-01

**Authors:** Anne M. Hocking, Jane H. Buckner

**Affiliations:** Center for Translational Immunology, Benaroya Research Institute at Virginia Mason, Seattle, WA, United States

**Keywords:** genetic variants, autoimmunity, immune tolerance, HLA, PTPN2, PTPN22, INS-VNTR, PADI

## Abstract

Genetic variants associated with susceptibility to autoimmune disease have provided important insight into the mechanisms responsible for the loss of immune tolerance and the subsequent development of autoantibodies, tissue damage, and onset of clinical disease. Here, we review how genetic variants shared across multiple autoimmune diseases have contributed to our understanding of global tolerance failure, focusing on variants in the human leukocyte antigen region, PTPN2 and PTPN22, and their role in antigen presentation and T and B cell homeostasis. Variants unique to a specific autoimmune disease such as those in PADI2 and PADI4 that are associated with rheumatoid arthritis are also discussed, addressing their role in disease-specific immunopathology. Current research continues to focus on determining the functional consequences of autoimmune disease-associated variants but has recently expanded to variants in the non-coding regions of the genome using novel approaches to investigate the impact of these variants on mechanisms regulating gene expression. Lastly, studying genetic risk variants in the setting of autoimmunity has clinical implications, helping predict who will develop autoimmune disease and also identifying potential therapeutic targets.

## Introduction

**Figure 1:**
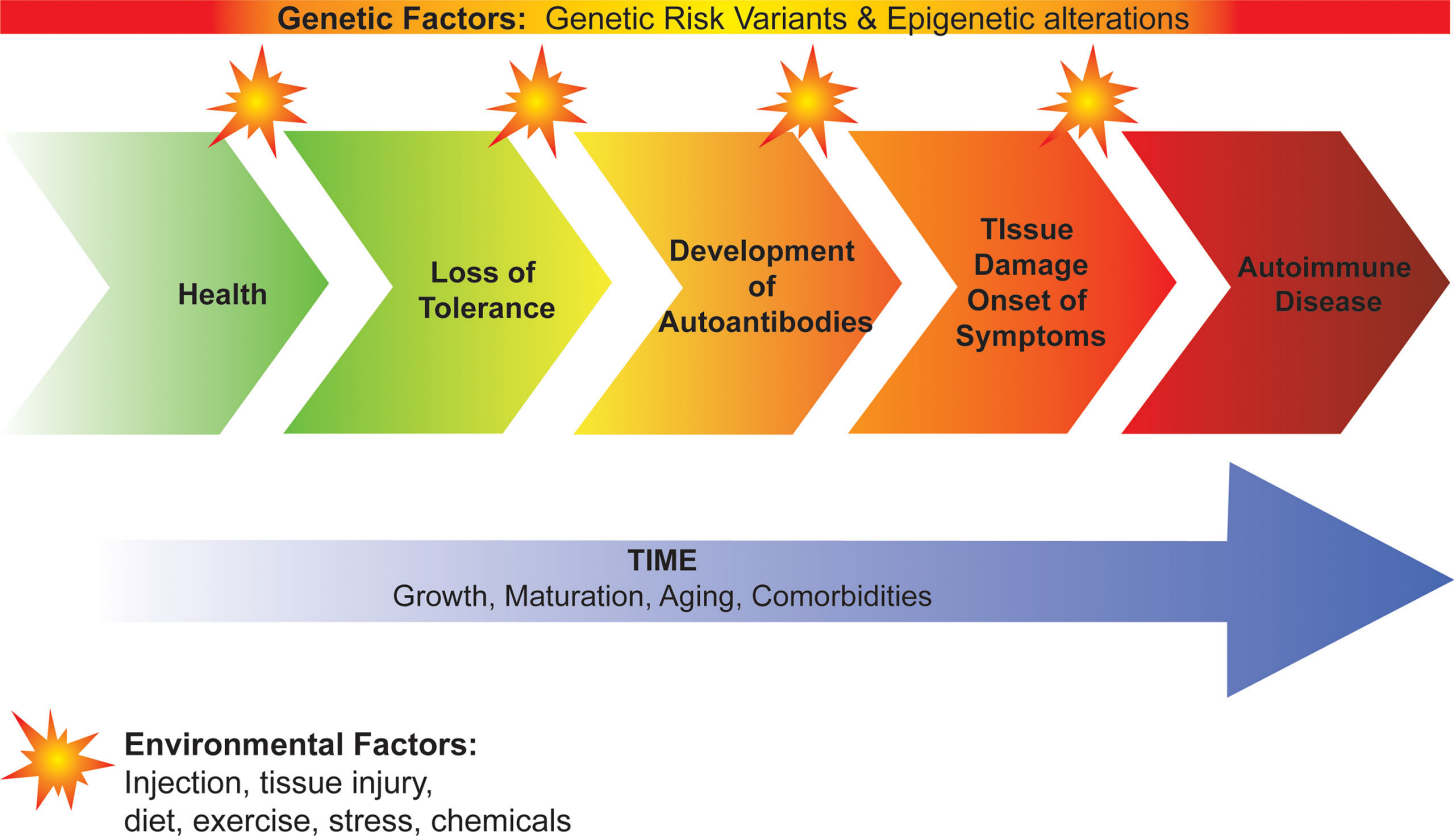
Development of autoimmunity and progression to autoimmune disease. Genetic factors predispose to loss of immune tolerance and the subsequent development of autoantibodies, tissue damage, and onset of clinical disease. Environmental factors act as triggers that initiate and promote disease progression. Growth, maturation, aging and comorbidities contribute to the rate of progression from loss of tolerance to autoimmune disease.

Development of autoimmunity and progression to autoimmune disease occurs on a continuum with the complex interplay of genetic factors and environmental factors over time (**Figure 1**). Genetic risk variants and epigenetic alterations predispose to loss of immune tolerance and the subsequent development of autoantibodies, tissue damage, and onset of clinical disease. Environmental factors are less understood but are thought to act as triggers that initiate and promote disease progression. To date, viral infection, tissue injury, diet, and stress have all been implicated in this process suggesting that there may be a “threshold effect” involving multiple triggers rather than a single trigger for autoimmunity. Time is also important with growth, maturation, and aging tuning the rate and direction of disease progression. In this review, we focus on the role of genetic variants, specifically how they contribute to failed immune tolerance in autoimmunity. We describe how they have enabled us to identify the molecular and cellular mechanisms underlying immune tolerance. We also provide an update on how genetic variants have helped predict disease development and have facilitated the identification of new therapeutic targets for treatment and prevention of autoimmune disease, including in the setting of personalized/precision medicine.

## Role of genetics in development of autoimmunity

Immune tolerance is defined as the state of unresponsiveness to molecules that have the potential to induce an immune response and ensures that the immune system does not mount a response against self-antigens. Importantly, failure of tolerance contributes to induction of autoimmunity (reviewed in (1)). Tolerance is achieved through both central and peripheral tolerance mechanisms (reviewed in (2)). Central tolerance occurs in the thymus for T lymphocytes and the bone marrow for B lymphocytes and acts primarily through negative selection by eliminating immature T and B lymphocytes that recognize self-antigens (2). Peripheral tolerance takes place after the T and B lymphocytes leave the primary lymphoid organs. Mechanisms through which tolerance is maintained in the periphery include: apoptosis, anergy, and regulatory T cell (Treg)-mediated suppression (1, 2).

**Figure 2:**
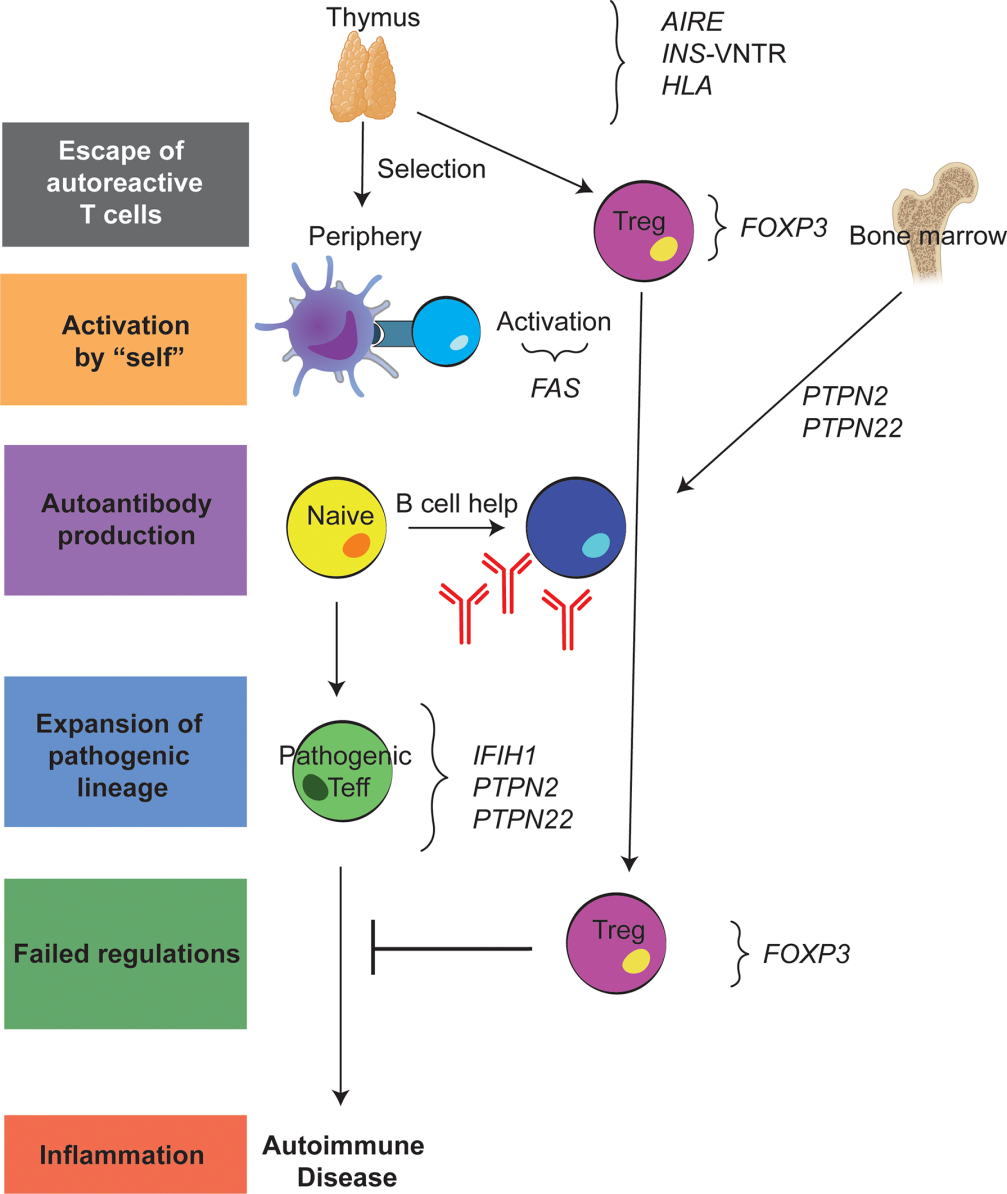
Genetic variants underlying failed immune tolerance and development of autoimmunity. Schematic showing where each of the discussed variants contribute to the loss of tolerance both in the thymus and the periphery.

Studies of monogenic disorders have been critical to understanding tolerance mechanisms (**Figure 2**). For example, autoimmune polyendocrine syndrome type-1 (APS-1) caused by mutations in the gene autoimmune regulator (*AIRE*) has provided key insight into central tolerance (3). Specifically, AIRE expression by medullary thymic epithelial cells promotes the display of tissue-specific antigens to developing T cells, a key step in negative selection of autoreactive T cells. This lack of central tolerance results in the development of multiple autoimmune diseases including type 1 diabetes (T1D), hypothyroidism, adrenal insufficiency, alopecia, and vitiligo. Conversely, studying both autoimmune lymphoproliferative syndrome (ALPS) and immune dysregulation, polyendocrinopathy, enteropathy, X-linked (IPEX) syndrome have increased our understanding of mechanisms of peripheral tolerance. ALPS caused by mutations in the first apoptosis signal receptor (*FAS*) gene demonstrates how failed apoptosis drives autoimmunity (4). IPEX syndrome, a multi-organ autoimmune disease from birth, caused by mutations in the transcription factor forkhead box P3 (*FOXP3*) that result in either a lack of Tregs or impaired Treg function, highlights the importance of Tregs in maintaining peripheral tolerance (5). Other rare monogenic forms of autoimmunity are also instructive including LPS-responsive and beige-like anchor protein (*LRBA)* deficiency, *CD25* deficiency and signal transducer and activator of transcription 3 (*STAT3)* gain-of-function, all of which impair Treg cell function (6, 7).

The majority of autoimmune diseases are polygenic and genome-wide association studies (GWAS) have identified genetic variants shared across multiple autoimmune diseases as well as variants unique to specific autoimmune diseases. Both types of variants have been informative providing insight into the signaling pathways and immune cell types involved in induction and maintenance of tolerance. The shared variants have been most instructive for our understanding of the global tolerance failure underlying autoimmunity whereas disease-specific variants have been more useful defining disease-specific immunopathology. However, defining the functional impact for both shared and disease-specific variants remains challenging since an individual variant may be expressed in multiple immune cell types at different developmental stages and/or at discrete phases of the immune response and may also be influenced by environmental factors. In addition, growing evidence indicates that genetic risk variants synergize with each other to promote autoimmunity (8, 9).

Understanding interactions between genetic risk variants is also important for the development of polygenic risk scores to predict disease susceptibility and disease progression and inform treatment options. In type 1 diabetes (T1D), these scores are being used to predict progression of islet autoimmunity and development of clinical disease in the at-risk population (10, 11). More recently, a combined risk score for T1D has been developed that integrates genetics, autoantibodies, and clinical factors (12). Genetic risk scores for predicting clinical outcomes are also being investigated in the setting of rheumatoid arthritis (RA) and systemic lupus erythematosus (SLE). In RA, a polygenic risk score has recently been developed to predict severity of radiographic progression (13) and in SLE, a high genetic risk score was associated with organ damage and renal dysfunction (14).

## Shared genetic variants across autoimmune disease

Over time multiple approaches have been undertaken to identify the genetic underpinnings of autoimmunity. These studies included targeted assessments of families with autoimmunity as well as case control association studies of candidate genes. These approaches successfully identified genes with a strong association with autoimmunity, including the HLA locus (reviewed in (15)) and the coding variant PTPN22 (16). The sequencing of the human genome and development of GWAS chip led to the ability to screen large numbers of affected and unaffected individuals. This allowed the identification of common variants that associated with risk for autoimmunity.

A key observation from the initial GWAS studies was that many genetic risk variants are shared across autoimmune diseases (17, 18). Notably, these shared genetic variants highlight the vital role of antigen processing and presentation, T cell activation, cytokine signaling, as well as innate sensing mechanisms in induction and maintenance of immune tolerance (19, 20). The breadth of information on the many genetic variants associated with autoimmunity is beyond the scope of this review. Instead, we will focus on two protein tyrosine phosphates, PTPN2 and PTPN22, due to their association with multiple autoimmune diseases and the evidence of their role in multiple aspects of immune tolerance, while also discussing human leukocyte antigen (HLA), the region most strongly linked to autoimmunity (**Figure 2**).

### Human leukocyte antigen class II alleles

The HLA region, a large polymorphic region on chromosome 6, encodes HLA Class II molecules, which function to present processed antigens to CD4 T cells (reviewed in (15)). The HLA Class II molecules are heterodimers composed of an alpha and beta chain expressed on the surface of antigen-presenting cells. Importantly the HLA Class II molecules contain a peptide-binding groove that allows formation of a trimolecular complex between the HLA Class II molecule, its bound peptide and the T cell receptor on T cells. The HLA Class II region has the strongest genetic association with human autoimmune diseases (15), underscoring the importance of antigen presentation for immune tolerance. HLA class II alleles are primarily associated with autoimmune diseases characterized by autoantibodies such as T1D, RA and SLE (15, 21–23). Notably, the HLA locus is highly polymorphic, and the allelic associations differ across autoimmune diseases suggesting that HLA is also involved in the tissue specificity of the immune response. Additionally, HLA alleles may be associated with protection as well as risk. T1D is an example where both are seen, HLA DR4, DR3 and DQ0302 are each associated with disease, whereas DQ0602 is protective (23). HLA alleles are also associated with disease characteristics. For example, HLA-DRB1 alleles encoding the shared epitope a “shared” motif (QKRAA, QRRAA or RRRAA in positions 70–74 of the DRB1 chain) that is found on DR1 and DR4 alleles associated with a distinct subset of individuals with RA, specifically those who have anti-citrulline antibodies (ACPA) or ACPA+ RA (reviewed in (24)). It is also important to note that the region linked to HLA risk on chromosome 6 includes additional genes with immunologic significance, and there is growing evidence that they too may impart risk for autoimmunity (25, 26).

### PTPN2

The *PTPN2* gene encodes protein tyrosine phosphatase non-receptor type 2, which has a regulatory role in a variety of signaling pathways including T cell receptor signaling, IL-2 signaling, and JAK/STAT signaling (27). There are three autoimmune disease-associated variants in the PTPN2 gene shared across T1D, Crohn’s disease, and RA: rs2542151 in the coding region and rs1893217 and rs478582 both in the non-coding region (27). The rs1893217 variant is associated with decreased PTPN2 mRNA levels in human T cells (28). Carriers of the rs1893217 variant also show impaired T cell responses to IL-2 as measured by pSTAT5 (28) and the rs478582 variant is associated with reduced stability of Tregs (29, 30). In murine models, PTPN2 expression is linked to T cell lineage commitment (31), proliferation and survival (32), and Treg stability (33). Yet as a broader understanding of the impact of altered PTPN2 expression is gained, its role in autoimmunity has extended beyond T cells. In murine models, PTPN2 has been shown to negatively regulate IL-21 signaling and B cell responses (34), in humans the PTPN2 risk variant rs1893217 is associated with the loss of B cell anergy (35). PTPN2 is also important in dendritic cell-mediated immune tolerance, partial depletion of PTPN2 in dendritic cells (DCs) results in spontaneous inflammation, altered immune cell composition, increased accumulation of conventional type 2 DCs (cDC2) in organs, and expansion of IFNγ-producing effector T cells (36). Notably, the variants of PTPN2 associated with autoimmunity are quite common (the minor allele frequency (MAF) of rs1893217 risk = 0.1196) and the increase in risk is modest (the odds ratios for T1D and Crohn’s are 1.3 and 1.25 respectively (27)) indicating that the risk variants contribute through modest alterations in multiple aspects of immune regulation.

### PTPN22

The rs2476601 variant in the coding region of the protein tyrosine phosphatase non-receptor type 22 (*PTPN22)* gene is one of the most strongly associated risk variants shared across autoimmune diseases including RA, T1D, and SLE (37). PTPN22 is notable for its role across multiple immune cell types including lymphocytes, natural killer (NK) cells, neutrophils, monocytes, macrophages, and DCs (37). In T and B cells, PTPN22 regulates antigen receptor signaling (38), making it a major focus of studies investigating its role in autoimmunity risk. The minor allele of the rs2476601 variant is associated with autoimmunity and has a thymine substituted for a cytosine at nucleotide 1858 (*PTPN22* C1858T) resulting in a change from arginine (R) to tryptophan (W) at amino acid position 620. This amino acid change results in modest alterations in the function of PTPN22 but importantly alters the character and function of immune cells. Examples of this include alterations in the composition of the B cell compartment and increases in polyreactive and autoreactive B cells in PTPN22^620W/W^ carriers, indicating a failure of B cell tolerance (39, 40). Notably, murine modeling of this variant recapitulates autoimmunity and confirms that a multiplicity of mechanisms is involved in this process (41, 42). Human studies have also shown that the rs2476601 variant influences T cell maturation including increased CD4 memory T cells (43) and an increase in Th1 cells (44). In addition, this variant impacts TCR signaling, although the jury is still out as to whether it is a gain- or loss-of-function mutation, and this is likely dependent on context (37). This PTPN22 variant has also been recently implicated in cDC2 homeostasis because expression of the orthologous polymorphism in mice lead to expansion of cDC2 (45). Thus, similar to PTPN2, the PTPN22 variant which is broadly associated with autoimmunity, likely does not confer risk through one pathway, but through a combination of modest alterations, that lead to failures in tolerance checkpoints in both T and B cell compartments and promote the development of pathogenic responses.

### Synergy between autoimmune disease-associated variants

Dissecting how autoimmune disease-associated variants interact with each other to promote susceptibility to autoimmunity is a critical next step for understanding how genetic variants contribute to the loss of tolerance. Although, this is challenging to do, it is possible using well-defined cohorts controlled for the genotypes of interest, and/or crossing knockin mouse models expressing the variants of interest. CRISPR/cas9 genome editing is also being utilized to express the variants of interest in primary human immune cells (46–48). Here, we highlight two studies investigating the interactions between genetic variants in the setting of autoimmune disease. The first analyzed a large cohort of individuals with RA and determined that there was synergistic interaction between the *PTPN22* s2476601 variant and the *HLA-DRB1* shared epitope alleles in participants who were positive for both antibodies to cyclic citrullinated peptides and antibodies to citrullinated α-enolase (49). Interestingly, the combined effect of the *PTPN22* s2476601 variant and the HLA-DRB1 shared epitope alleles was further enhanced by smoking (49), underscoring the importance of gene-environment associations for the development of autoimmunity. The second study crossed knockin mice to investigate the interaction between the rs1990760 variant in *IFIH1* and the rs2476601 in *PTPN22* (8). The *IFIH1* variant rs1990760 is associated with risk of T1D, SLE, RA, and multiple sclerosis (MS) (50) and results in an amino acid change from alanine to threonine at position 946 in the C-terminal of the interferon-induced helicase C-domain containing protein 1 (IFIH1 also known as MDA5). IFIH1 is a pattern recognition receptor for dsRNA that induces a type I interferon response to RNA viruses (51). In both humans and mice, the IFIH1 rs1990760 variant acts as a gain-of-function mutation that increases the interferon response (8). When both the *IFIH1* rs1990760 and *PTPN22* rs2476601 variants were introduced into a murine model of T1D, an additive effect was observed with increase in the rate and time to onset of diabetes (8). These studies are examples of the interaction across genetic variants and indicate such interactions may amplify disease risk.

### Variants unique to a disease reveal disease-specific immune alterations

There are also genetic variants that are only associated with a single autoimmune disease. Interestingly, these variants typically target a pathway or process that is unique to the underlying pathogenesis of disease such as the antigen targeted in autoimmunity. As noted above, the HLA locus is associated with many autoimmune diseases, but the associated alleles may differ- arguing that the link at this level may be specific to the autoantigen being targeted. Other variants that are disease-specific and associated with specific antigen targets include the insulin variable number of tandem repeats (*INS*-VNTR) variant associated with T1D and the peptidylarginine deiminase (*PADI*) 2 and 4 variants associated with RA.

### *INS*-VNTR

The polymorphic insulin gene variable number of tandem repeats (*INS-*VNTR) is associated with the proinsulin gene promoter region. Variants in this region, specifically the VNTR III haplotype, are associated with a 3- to 4-fold relative protection from diabetes (52). This haplotype is associated with elevated expression levels of proinsulin in the thymus (53, 54) and a decrease in the frequency of high avidity pro-insulin-specific CD4^+^ T cells in comparison to the diabetes susceptibility haplotype VNTR I (55). Thus, in a manner similar to the AIRE mutation that limits expression of self-antigens in the thymus, this genetic risk variant may act by specifically impeding the expression of pro-insulin in the thymus resulting in a tissue-specific failure of central tolerance which can contribute to the development of pathogenic proinsulin-specific T cells and ultimately the development of T1D.

### *PADI2* and *PADI4* variants

Genetic variants in *PADI2* and *PADI4* have been associated with ACPA positive-RA (56), although these associations appear to be strongest in Asian populations (57). The functional impact of these variants is still unclear yet the role of peptidylarginine deiminases (PADs) in RA makes this association of particular interest. *PADI2* and *PADI4* encode (PADs) 1 and 4 respectively, enzymes that catalyze the post-translation conversion of arginine to citrulline by calcium-dependent deamination (58). Given that ACPA are present in 80% of individuals with RA, PADs are likely to play a central role in disease pathogenesis due to their ability to generate citrullinated proteins. This is further supported by the presence of PAD2 and PAD4 in the synovial fluid of patients with RA (59–61). In addition, PADs are involved in immune cell processes implicated in autoimmunity, including neutrophil net formation (netosis) (62) and anti-PAD4 antibodies have been detected in patients with RA and are associated with disease severity (63). Also intriguing is a recent study reporting an association with *PADI4* variants in Caucasian individuals who smoked and carried risk alleles for both HLA-DRB1*04 and PTPN22 (64). A potential explanation for this synergy is the discovery that PTPN22 interacts with and inhibits PAD activity, but the PTPN22^620W^ risk variant (rs2476601) disrupts this interaction leading to enhanced citrullination and netosis (65).

## Future directions

The next frontier in the field of autoimmune disease-associated genetic variants is understanding the functional impact of non-coding variants located in regulatory regions of the genome. New discovery opportunities are now possible due to advances in approaches to interrogate the 3-dimensional architecture of the genome including chromatin conformation capture techniques and increasingly sophisticated profiling methods integrating epigenetics, transcriptomics, and proteomics. In addition, there have been substantial improvements in the assays used to elucidate the function of a variant with massively parallel reporter assays (MPRA) and CRISPR-Cas genome editing facilitating high throughput screening. These approaches are now being applied to autoimmune disease-associated variants. A CRISPR activation screen identified a risk variant in an enhancer region of the IL2RA gene (47) and more recently MPRA was used to prioritize approximately 18,000 autoimmune disease associated-variants based on how they perturb regulatory elements in T cells (66). Expression quantitative trait (eQTL) analysis has also been helpful in linking non-coding variants to nearby genes (67). Two recent studies applying single cell eQTL analysis to T cells highlighted the importance of both activation state and cell type on the effects of autoimmune disease-associated variants (68, 69).

Another priority is identifying genetic variants that are associated with either disease progression or response to treatment. This is an emerging field, but the power of this approach has been demonstrated by a study comparing good and poor prognosis in Crohn’s disease (70). Notably, variants were identified that were specifically associated with prognosis rather than susceptibility (70). Screening for genetic variants that influence response to therapy has also been limited, but there have been some genetic associations identified for response to TNFα blockade in RA. The biggest challenge for any of these studies is defining the cohort given the heterogeneity with respect to stage of disease and the therapies administered. This type of work will require strong collaborative efforts to assess clinical outcomes for large numbers of patients with studies of mechanistic outcomes; large undertakings with important potential to improve the way we provide healthcare to individuals at risk for and with autoimmune diseases.

## Clinical implications

Identification of genetic variants has been important for the development of immunotherapies aimed at achieving immune tolerance. For example, knowing the HLA haplotype is crucial for many antigen-specific therapies including peptide immunization and engineered Treg cell therapy. Genetic variants in the IL-2 signaling pathway such as those in *IL2RA* and *PTPN2* also need to be considered for IL-2-mediated therapy. Likewise for the IL-6 pathway where single nucleotide polymorphisms in the IL-6 receptor may influence the response to IL-6 blockade therapies. Tyk2 inhibitors are now in clinical trials, with initial encouraging results in psoriasis with the potential to be extended to other autoimmune diseases, particularly those associated with protection from the loss-of-function variant, including SLE and MS (71). Collectively, these studies underscore the value of autoimmune-associated genetic variants for development of personalized/precision medicine for the prevention and treatment of autoimmune disease.

## Author contributions

AH collected literature and wrote the review. JB conceived, collected literature, and wrote the review. All authors contributed to the article and approved the submitted version.

## Funding

This work was supported by National Institutes of Health (NIH) grants DP3 DK097672, R21 AR073508 and R01 AI132774 to JB.

## Acknowledgments

The authors would like to thank Dr. Taylor Lawson of the Benaroya Research Institute Scientific Writing Group for assistance with figure preparation.

## Conflict of interest

J.H.B. is a Scientific Co-Founder and Scientific Advisory Board member of GentiBio, a consultant for Bristol-Myers Squibb and Hotspot Therapeutics, and has past and current research projects sponsored by Amgen, Bristol-Myers Squib, Janssen, Novo Nordisk, and Pfizer. She is a member of the Type 1 Diabetes TrialNet Study Group, a partner of the Allen Institute for Immunology, and a member of the Scientific Advisory Boards for the La Jolla Institute for Allergy and Immunology and BMS Immunology.

The remaining author declares that the research was conducted in the absence of any commercial or financial relationships that could be construed as a potential conflict of interest.

## Publisher’s note

All claims expressed in this article are solely those of the authors and do not necessarily represent those of their affiliated organizations, or those of the publisher, the editors and the reviewers. Any product that may be evaluated in this article, or claim that may be made by its manufacturer, is not guaranteed or endorsed by the publisher.
